# Impact of thickness variation on structural, dielectric and piezoelectric properties of (Ba,Ca)(Ti,Zr)O_3_ epitaxial thin films

**DOI:** 10.1038/s41598-018-20149-y

**Published:** 2018-02-01

**Authors:** Valentin Ion, Floriana Craciun, Nicu D. Scarisoreanu, Antoniu Moldovan, Andreea Andrei, Ruxandra Birjega, Corneliu Ghica, Fabio Di Pietrantonio, Domenico Cannata, Massimiliano Benetti, Maria Dinescu

**Affiliations:** 1 National Institute for Laser, Plasma and Radiation Physics, 409 Atomistilor, 077125 Magurele, Romania; 2 CNR-ISC, Istituto dei Sistemi Complessi, Area della Ricerca di Roma-Tor Vergata, Via del Fosso del Cavaliere 100, I-00133 Rome, Italy; 3 National Institute of Materials Physics, 105 bis Atomistilor, 077125 Magurele, Romania; 4 CNR-IMM, Institute for Microelectronics and Microsystems, Via del Fosso del Cavaliere 100, I-00133 Rome, Italy

## Abstract

It is shown that the dielectric and piezoelectric properties of Ba(Ti_0.8_Zr_0.2_)O_3_-x(Ba_0.7_Ca_0.3_)TiO_3_ (x = 0.45) (BCTZ 45) epitaxial thin films have a nontrivial dependence on film thickness. BCTZ 45 epitaxial films with different thicknesses (up to 400 nm) have been deposited on SrTiO_3_ by pulsed laser deposition and investigated by different combined techniques: conventional and off-axis X-ray diffraction, high resolution transmission electron microscopy and dielectric and piezoforce microscopy. The changes occurring in epitaxial films when their thickness increases have been attributed to a partial relaxation of misfit strain, driving the induced tetragonal symmetry in very thin films to the original rhombohedral symmetry of the bulk material in the thickest film, which influences directly and indirectly the dielectric and piezoelectric properties.

## Introduction

In the last decades, focus on multifunctional piezoelectric materials has been shifted from traditional lead zirconate titanate-based ceramics to lead-free ceramics, due to the restrictions in use for materials with health and environmental risks. Some of the most attractive among the different alternatives of lead-free piezoelectric materials are (Ba,Ca)(Ti,Zr)O_3_ (BCTZ) solid solutions^1–4^. These have a very versatile composition, obtained as a combination of two end members, usually in the form (1−x) Ba(Ti_0.8_Zr_0.2_)O_3_-x(Ba_0.7_Ca_0.3_)TiO_3_ (BCTZ 100x), with a perovskite structure similar to that of the parent BaTiO_3_ ferroelectric perovskite. Doping with Ca on A- and Zr on B-sites of the perovskite structure allows obtaining various compositions with different properties, starting from relaxors with high dielectric constant for high energy density storage devices to ferroelectrics with high piezoelectric constant for actuators, energy harvesting, solid state cooling devices etc. BCTZ displays a nearly morphotropic phase boundary (MPB) at x ≈ 0.50 where the dielectric, ferroelectric, and piezoelectric properties are maximized^1–5^. Thus in refs^6,7^ Wu *et al*. have evidenced the maximization of dielectric and piezoelectric/ferroelectric properties of (Ba_0.85_Ca_0.15_)(Ti_1-y_Zr_y_)O_3_ when Zr content is y = 0.1 (which is equivalent to x = 0.5 in the two-end member formulation).

Different studies reported a phase diagram with a rhombohedral (R)–tetragonal (T) MPB separated by an intermediate orthorhombic (O) phase, as in BaTiO_3_^3,4,8^. In a previous work^9^ we have investigated the role of the intermediate O phase in enhancing the transverse instability and the elastic compliance at the MPB between R and T phases. The existence of an intermediate phase allows polarization to continuously rotate between the tetragonal and rhombohedral phases^10^. Alternatively, it has been evidenced that the intermediate phase behaves on microscopic scale as a nanotwinned phase with dense mobile walls leading to a high piezoelectric response due to the easy wall motion^11^.

Fabrication of thin BCTZ films by different techniques has been previously reported^12–15^. Thus, in a previous work, we have evidenced the relationship between nanostructure and dielectric properties of BCTZ epitaxial thin films grown on different substrates^16^. We have also deposited BCTZ thin films on flexible polymeric substrates and demonstrated their biocompatibility for use in biomedical applications^17^. However a thoroughfull investigation of the dependence of epitaxial BCTZ films properties on their thickness has been not yet made. Nevertheless, there are many previous papers which report the effect of thickness variation on structure and properties of ferroelectric/piezoelectric materials. Thus ref.^18^ reviews the properties of epitaxial ferroelectric thin films and their dependence on thickness, besides other factors. The characteristics of domain structure of films grown on different substrates and the relationship with the elastic misfit strain are presented. The dependence of structure and functional (ferroelectric/magnetic) properties of other lead-free (BiFeO_3_) thin films on thickness are discussed in refs^19,20^. Thickness-dependent properties of relaxor-PbTiO_3_ ferroelectric single-crystal layers are presented in ref.^21^.

In this work we present results obtained on epitaxial BCTZ thin films with different thicknesses and we report for the first time the dependence of their structural, dielectric and piezoelectric properties on thickness obtained by employing different correlated investigation techniques: conventional and off-axis X-ray diffraction (XRD), transmission electron microscopy (TEM), high resolution TEM (HRTEM), dielectric spectroscopy and piezoforce microscopy (PFM).

## Results and Discussion

### Structural characterization of thickness dependent BCTZ thin films

**Figure 1:**
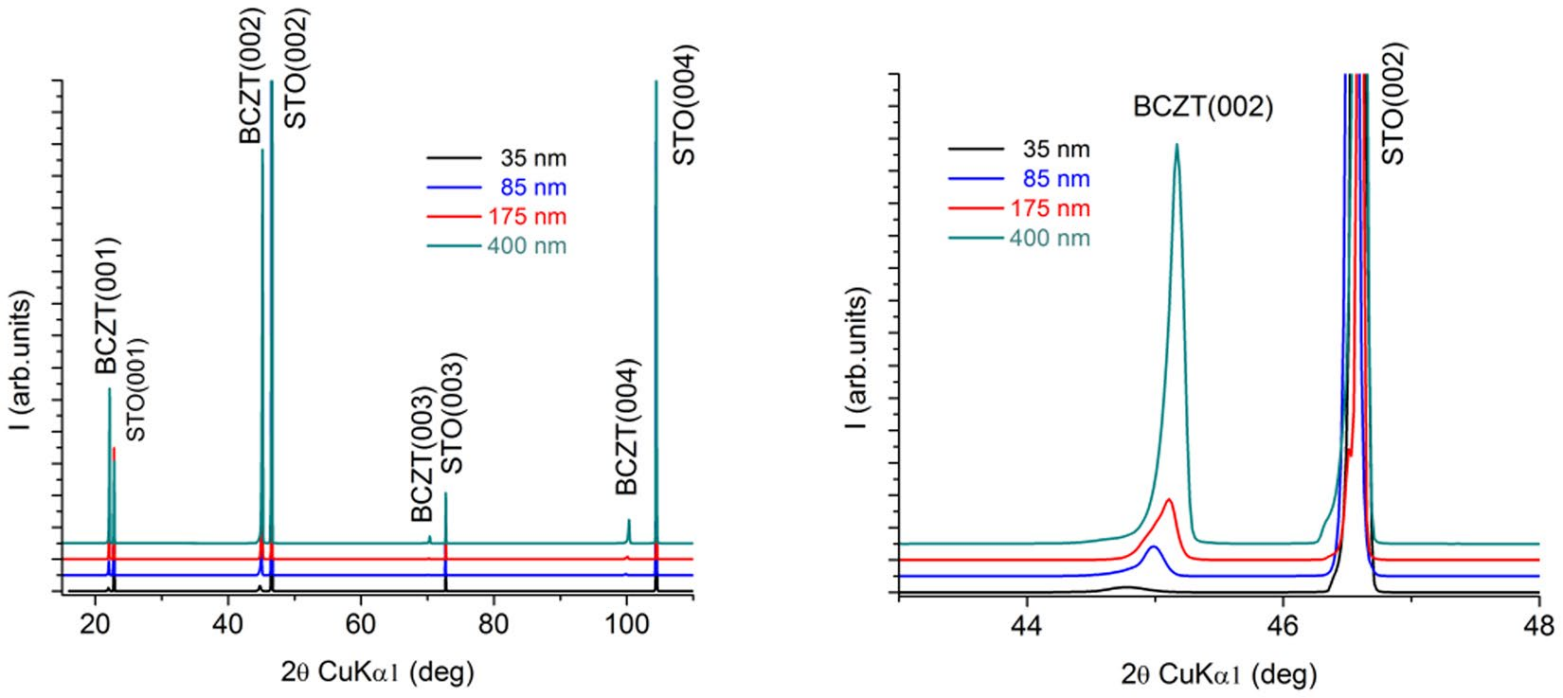
(**a**) XRD patterns of the films with different thicknesses; (**b**) enlargement of the zone near (002) peak.

The XRD patterns of the films with different thicknesses: 35, 85, 175 and 400 nm are displayed in Fig. 1. The patterns exhibit only (00l) peaks, evidencing the highly c-axis oriented growth of the BCTZ films, in spite of the large misfit (about 2.9%) between the pseudocubic lattice parameter of the BCTZ target (a = 4.0176 A) and the STO (001) substrate (a = 3.905 A). The enlargement of the zone near the (002) peak shows that the intensities are proportional with the thickness of the films, as expected. Moreover, while the structure of BCTZ 45 targets is rhombohedral, the epitaxial films show tetragonal structure, due to the constraining imposed by the substrate.

**Figure 2:**
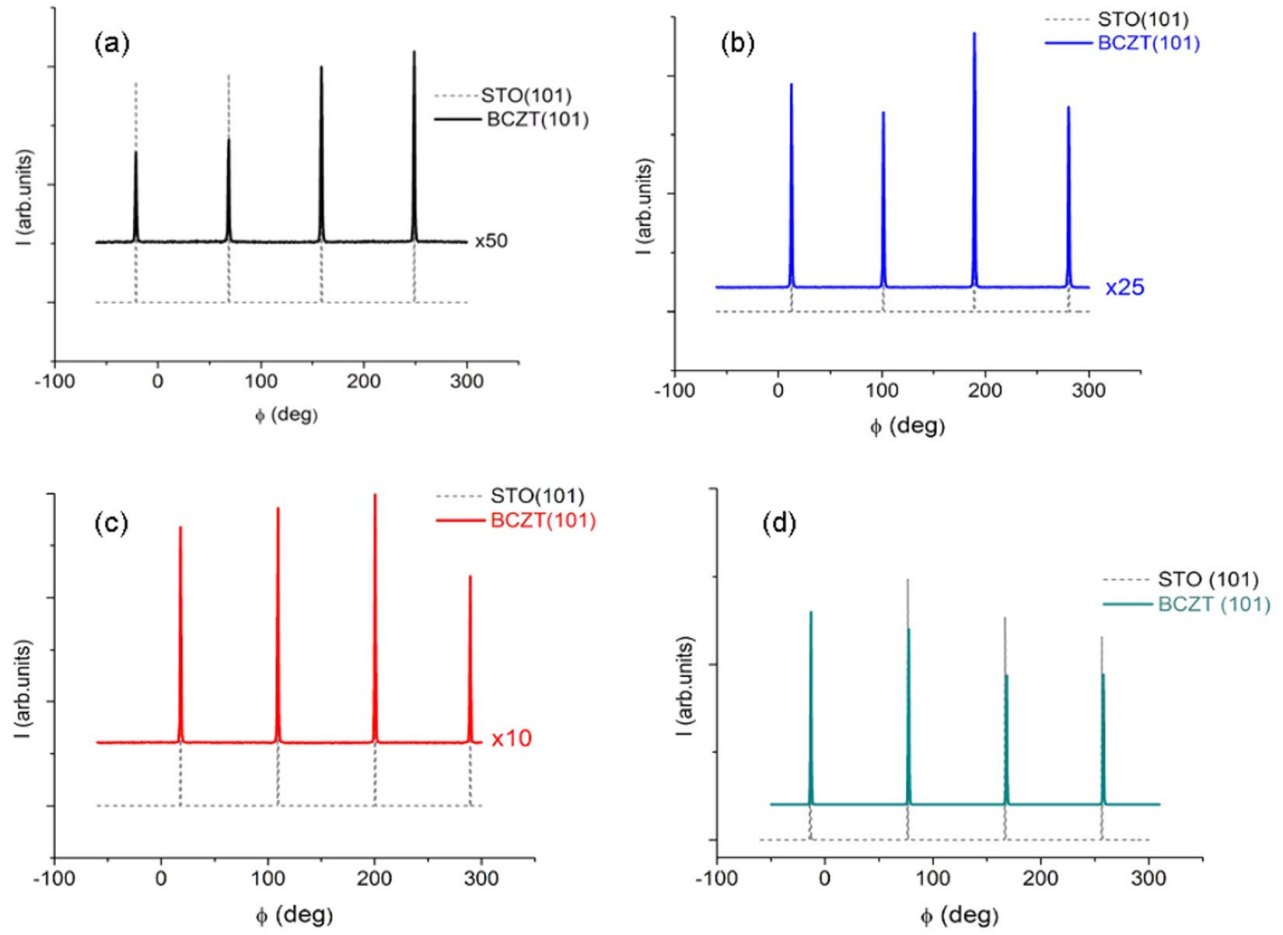
X-ray Φ-scans around (101) plane of BCTZ films with different thicknesses: (**a**) 35 nm; (**b**) 85 nm; (**c**) 175 nm; (**d**) 400 nm.

X-ray Φ-scans were performed for the characterization of the in-plane alignment of the BCTZ film around its (101) plane (Fig. 2). A fourfold symmetry is clearly seen, which is an indication of the cube-on-cube epitaxial growth of the films. No other peaks are observable in the intervals of these four peaks, showing that the BCTZ films are epitaxial with [100]BCTZ//[100]STO, [001]BCTZ//[001]STO for all the thicknesses, including 400 nm. It is remarkable that even at this relatively large thickness the films preserve their epitaxial characteristics, despite also the high mismatch strain. This is due to the careful selection of deposition parameter values, which allows the epitaxial lattice growth, but further studies are necessary to elucidate these aspects. Regarding these aspects, it is worth pointing out that the morphological features of the films surfaces do not seem to vary significantly with the thickness of the films, as revealed by the Atomic Force Microscopy images presented in Fig. S1(a),(b),(c) and (d) - Supplementary Information file. In spite the rare apparition of some droplets formations on the surface of the films, common feature for films produced by PLD technique, the aspect of the films surfaces does not change with thickness. However, a small variation of the roughness values can be seen between the 35 nm thick film- root mean square (RMS) value of 1.7 nm (Figure S1(a)) and the thicker 85 and 175 nm films with RMS values of 0.8 nm and 0.7 nm (Figure S1(b) and (c)), respectively. The higher RMS value of the thinnest film can be associated with the high mismatch strain level in the film.

**Figure 3:**
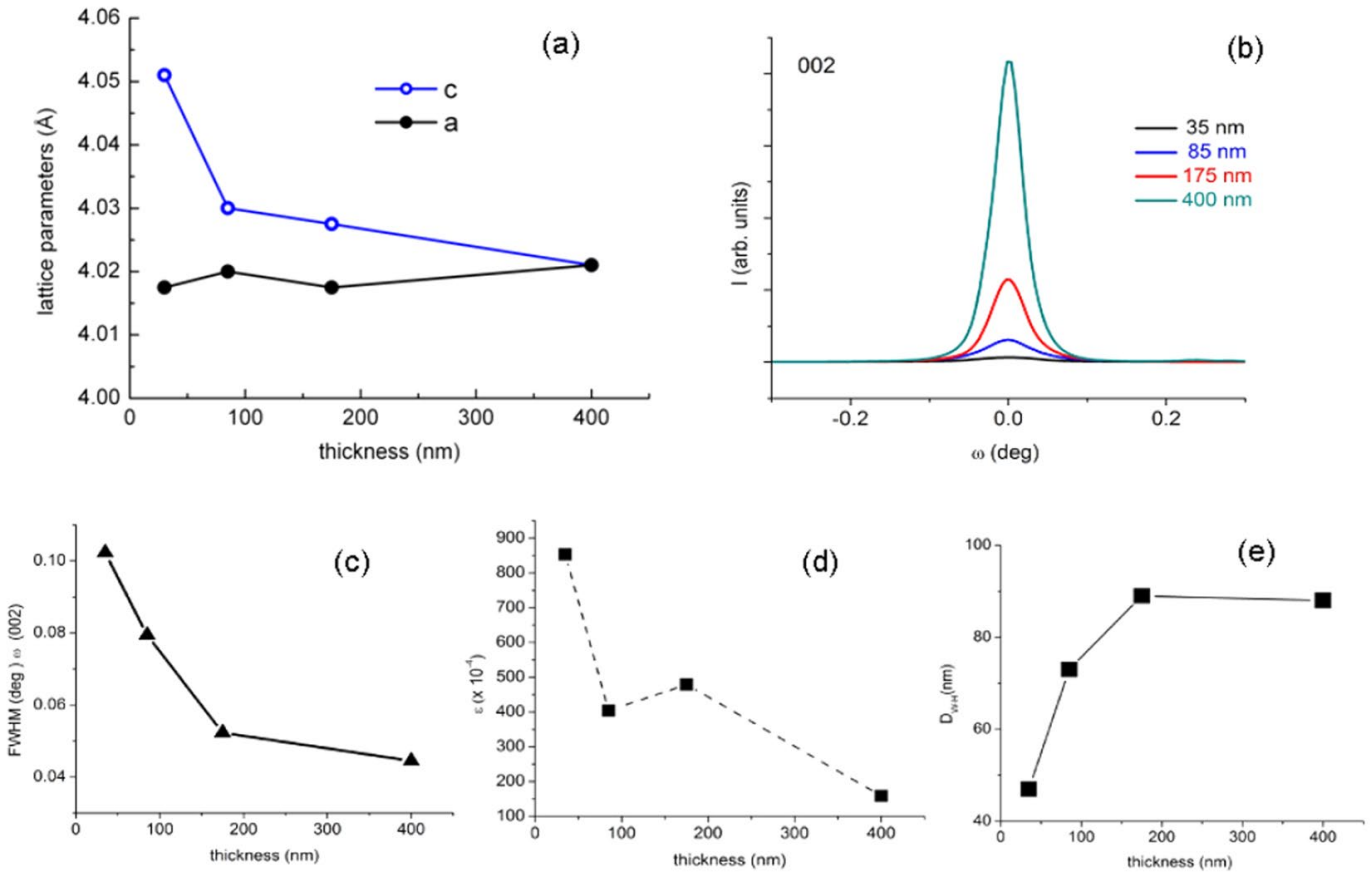
(**a**) the out-of-plane and in-plane lattice parameters for films with different thicknesses; (**b**) rocking curves for the different films; (**c**) mosaicity expressed via the FWHM of the peaks; (**d**) microstrain dependence on thickness; (**e**) crystallite size i. e. the coherent crystallographic domain size dependence on thickness.

The out-of-plane and in-plane lattice parameters, shown in Fig. 3a, were determined using conventional and off-axis diffraction, as described in our previous papers^16,22^. The evolution of these parameters evidences a strong anisotropy for the very thin film (35 nm); however it decreases rapidly when the thickness approaches 100 nm. For the thickest film (400 nm) the out-of-plane and in-plane parameters are nearly equal and similar to the bulk.

The rocking curves shown in Fig. 3(b) evidence the high crystallinity of the films. The mosaicity expressed via the FWHM of these peaks improves with thickness increasing (Fig. 3c).

The microstrain values (Fig. 3d) and the crystallite size i.e. the coherent crystallographic domain size (Fig. 3e) has been estimated from the Williamson-Hall plots^23^. It can be observed that there is a marked decrease of microstrain and a strong increase of the crystallite size when the film thickness increases. Generally the films show a slight asymmetric profile shape, discernible especially for the high order Miller indices reflections, as can be seen from Fig. S2. In the Supplementary Information file, the XRD diffraction patterns in logarithmic scale are presented for the (002) and (004) reflections domains- Figure S2. We related this observation mostly to an inhomogeneous in-plane compressive strain contribution to the broadening of these reflections due to a large film/substrate lattice mismatch. The shift in the profile’s asymmetry included in Fig. S2, was quantified by the parameter κ = *2θ*_max_−*θ*_*mean*_, were *2θ*_*max*_ and *θ*_*mean*_ are the Bragg-angles of the maximum and the mean of the profile, respectively^24^. Chen *et al*. evidenced this effect for epitaxial grown ZnO film on c-plane sapphire exhibiting an extremely large lattice mismatch^25^. They associated this effect also with a large thermal mismatch affecting the post-growth cooling period. Furthermore, they related the presence of this tail in the *θ*−2*θ* scans caused by inhomogenous strain in the interface region with the mosaicity of the film. Although the ordering along the growth direction is excellent the corresponding rocking curve is not a typical Gaussian curve. The line shape consists of two contributing overlapping peaks, a broad one and a sharp central peak^25^. Similarly, the rocking curves of the (002) reflection of the BCZT films with different thickness presented in logarithmic scale in the Fig. S3 (Supplementary Information), reveal a similar trend in particular for 35 nm film. The result is due probably due to the higher contribution of interface region versus the epilayer. The effect is attenuated in the 2*θ*/*θ* scans of this film for which no asymmetry was observed by the significant contribution of its smaller crystallite sizes in the reflections broadening. However, the discussion could not exclude the eventually distortion symmetry effects bearing in mind the proximity of the BCZT 45 composition to the MPB.

**Figure 4:**
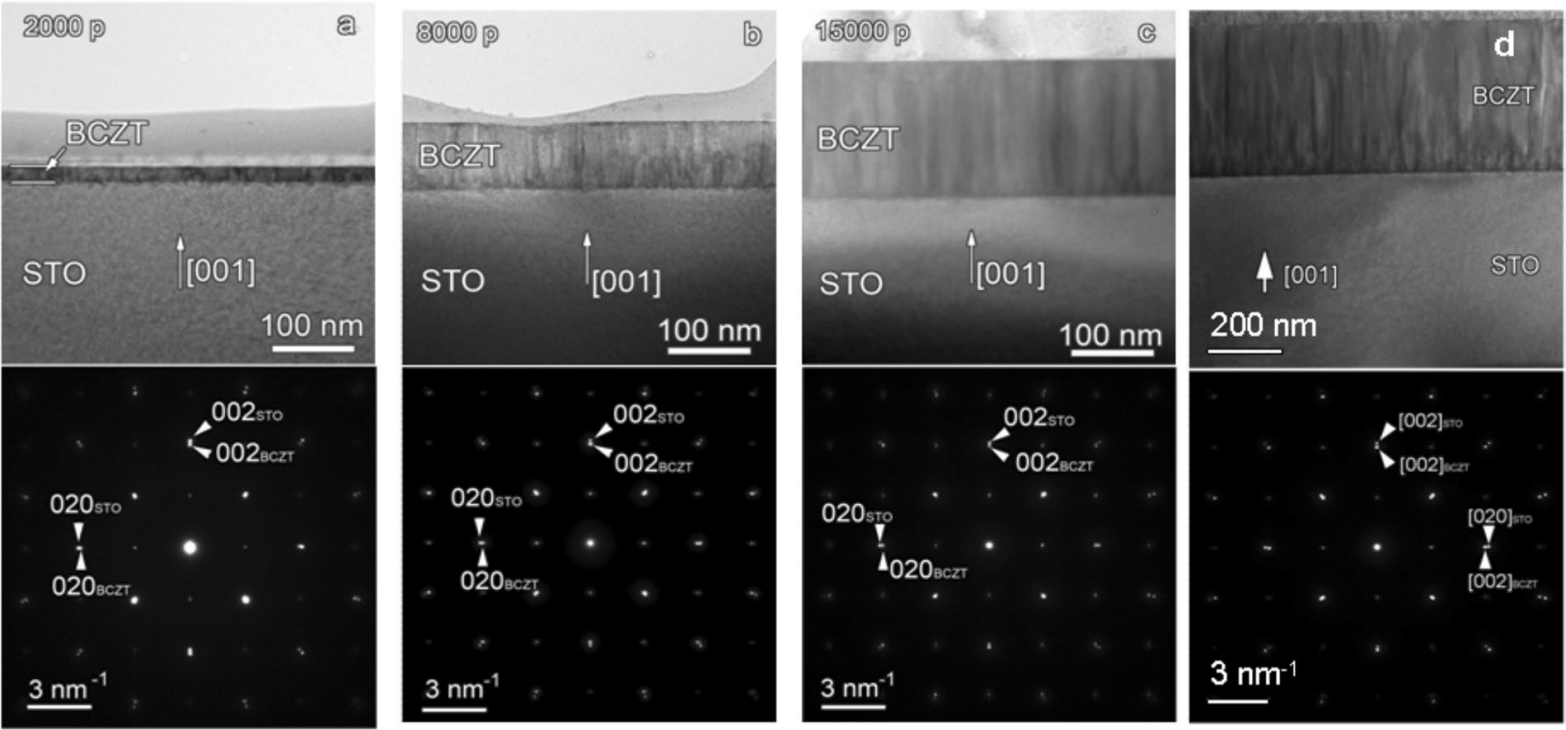
Low-magnification TEM images and the corresponding SAED patterns of BCTZ/STO films with thicknesses 35 nm (**a**), 85 nm (**b**), 135 nm (**c**) and 400 nm (**d**).

Figure 4 displays cross-section low magnification TEM images and the corresponding selected area electron diffraction (SAED) patterns of BCTZ/STO thin films with thickness of 35 nm, 85 nm, 175 nm and 400 nm, respectively. The layers are compact, smooth, showing no droplets on the surface. A columnar growth is visible for all films. The columns seem to have a width around 10 nm and a length which in some cases extends along all the film thickness. The SAED patterns certify the epitaxial growth for all the BCTZ layers. The splitting of the diffraction spots, noticeable especially in the case of the higher order Miller indices, indicates the tetragonality of the BCTZ layer and the lattice mismatch with respect to the STO substrate. The TEM images present a diffraction contrast associated to the elastic strain within the films, along the interface with the substrate and at the contact between the growth columns.

**Figure 5:**
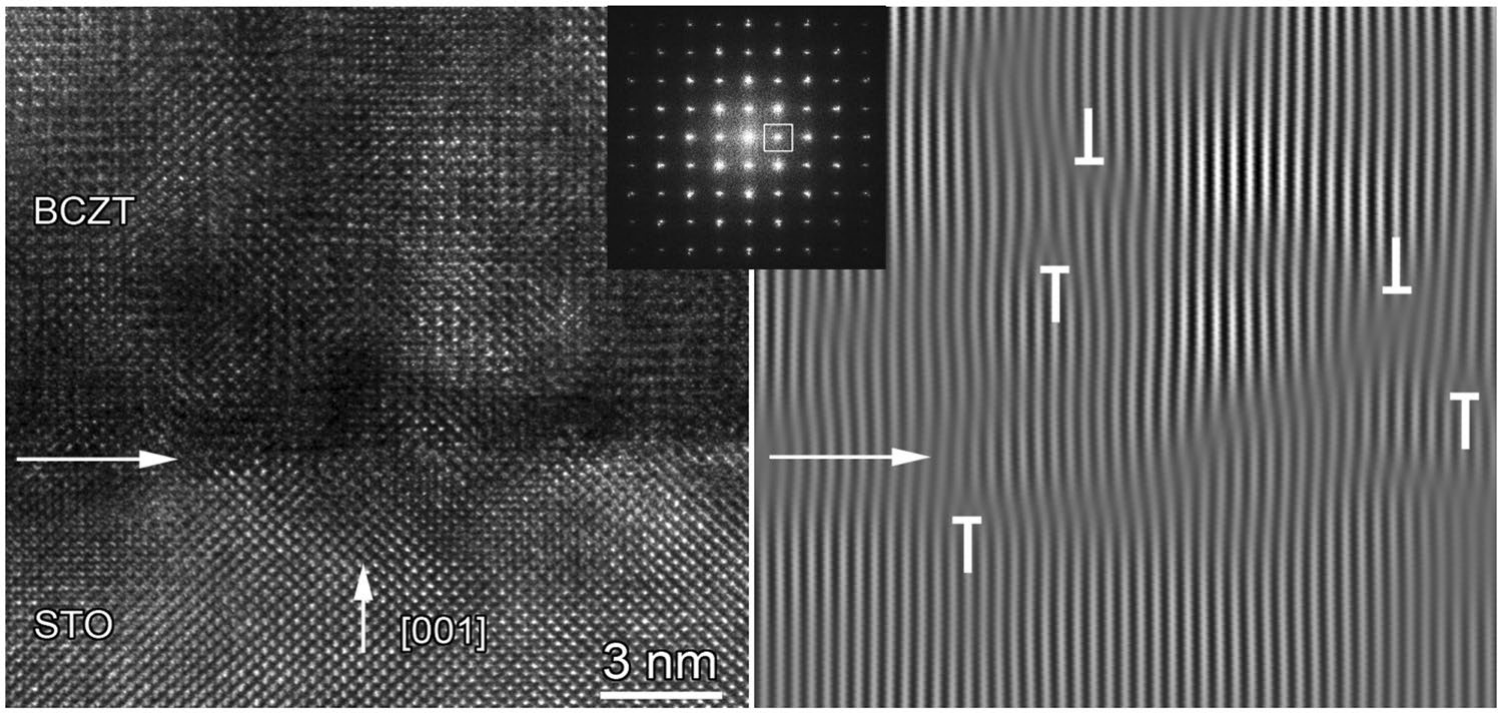
A typical HRTEM image of the interface between the BCTZ layers and the STO (001) substrate, together with the FFT image.

A typical HRTEM image of the interface between the BCTZ layers and the STO (001) substrate is shown in Fig. 5. The white arrow marks the trace of the film-substrate interface. A slight variation of the HRTEM contrast, especially close to the interface, indicates the presence of remnant strain due to the lattice mismatch. The epitaxial growth is also pointed by the fast Fourier transform (FFT) of the HRTEM image. By selecting the 010 peak (white square on the FFT) and performing the inverse Fourier transform, the Bragg filtered image reveals the presence of some dislocations (supplementary half-planes marked by T-signs) along the interface with the substrate, but also inside the film.

An important issue which might directly influence the local structural distortions is the film composition with respect to the nominal values in the PLD target and the compositional homogeneity of the thin films, especially that the chosen composition involves a large number of cations. These two aspects have been investigated by EDS measurements performed both in TEM and STEM modes, presented in the Supplementary Information file. A typical EDS spectrum obtained on a cross-section sample in the TEM mode on the 85 nm thick BCTZ layer using a spot size of the same order of magnitude as the film thickness is presented in Figure S4- Supplementary Information file. The spectrum contains the X-ray signatures corresponding to Ba, Ca, Ti, Zr and O. The Cu lines are due to the copper TEM ring used as support for the thin specimen. The quantitative analysis of this spectrum is neither straightforward nor entirely reliable due to the overlap between the Ti Kα line at 4.510 keV and Ba Lα line at 4.460 keV. The 50 eV separation between the two spectral lines is below the instrumental spectral resolution (ca. 125 eV for the Mn Kα line at 5.894 keV). In order to diminish the measurement errors for the quantitative analysis of Zr and Ba we used the Kα lines at 15.7 keV and 32.2 keV, respectively. Considering the cautions mentioned above, the effective thin films composition cannot be evaluated with respect to the nominal values in the target’s structure. We extended our compositional investigations by analyzing the same specimen in the STEM mode in order to reveal some possible local compositional fluctuations. The STEM ADF image of the BCTZ layer is presented in Figure S5. Our study on the chemical composition of the BCTZ layers indicate a rather homogeneous spatial distribution of the chemical elements in the layers. Although the standardless EDS technique that we used is semi-quantitative and despite the difficulties related to the signals overlap, our investigations provide an indication as to the compositional uniformity which, corroborated with the STEM-ADF contrast, indicate a slight increase of the Ti concentration in close proximity to the interface (band of ca. 10 nm). By extrapolating, we may therefore consider that the STEM contrast variations noticed inside the BCTZ layer might be associated with slight compositional variations that could induce local structural distortions, given that the chosen nominal stoichiometry is close to the MPB.

### Dielectric permittivity and piezoelectric response of BCTZ thin films with different thickness

The in-plane dielectric permittivity and loss have been determined from measurements on different IDT, as described in our previous paper^16^.

**Figure 6:**
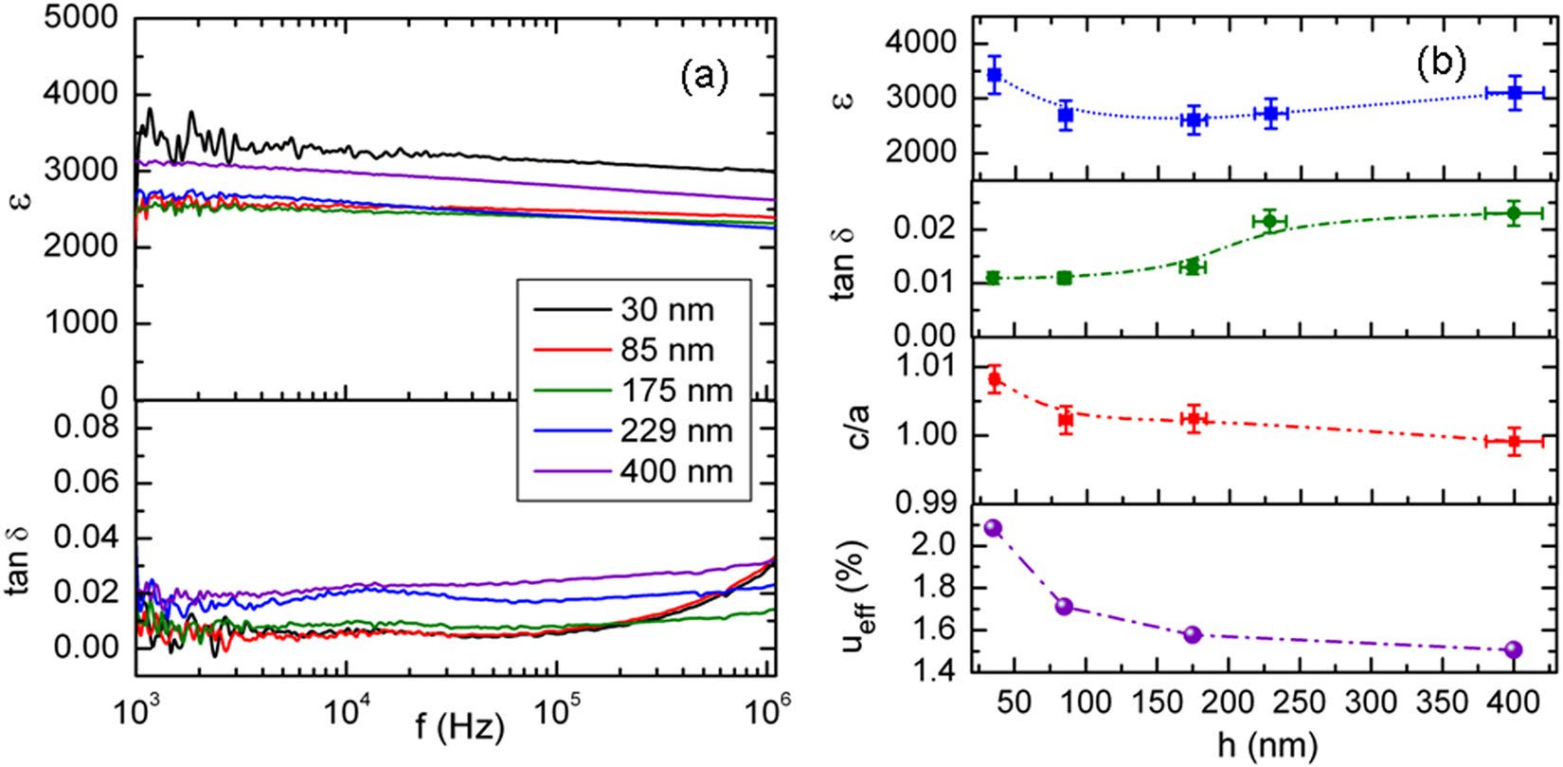
(**a**) Dielectric permittivity and loss variation with frequency at room temperature for films of different thicknesses; (**b**) Dependence of dielectric permittivity (at 1 kHz), dielectric loss tangent (at 1 kHz), tetragonality ratio and effective strain on film thickness.

Figure 6(a) shows the calculated dielectric constant and tan δ for all the films (plus a supplementary sample, with thickness 229 nm, on which only dielectric measurements have been performed), in the frequency range 1 kHz-1 MHz. It can be observed that the obtained values are nearly constant over three decades. To better display the variation with thickness, the values obtained at 1 kHz have been represented in Fig. 6(b) as a function of film thickness, together with the tetragonality factor c/a. As it can be observed, the in-plane dielectric constant reaches the highest value (about 3400) for the film with the lowest thickness (35 nm), which has also the highest tetragonality, due to the strong epitaxial strain. The high value of the dielectric constant is associated with the dipole and polarization increase due to the higher tetragonality. The dielectric loss is small (nearly 1%) although it starts to slightly increase above 100 kHz. When the film thickness increases, namely in the range 85–175 nm, the dielectric constant decreases, varying in the range 2600–2700. This is due to the relaxation of epitaxial strain and consequent decreasing of polarization.

However, for much thicker films (about 400 nm) the in-plane dielectric permittivity does not continue this tendency but increases up to 3100, which is explained by the near-surface dielectric softness of thicker films. Moreover, in all the thickness range, the values of the dielectric permittivity are much higher than in corresponding bulk materials, due to the previously evidenced peculiar nanostructure in partially relaxed epitaxial films^16^ and the high crystallinity of the columnar growth in epitaxial films.

In epitaxial thin films the misfit strain is controlled by selecting the appropriate substrate. However, the internal stress in the film is also dependent on the film thickness due to the relaxation of the lattice mismatch induced stress by the occurrence of dislocations and other lattice defects during growth^26,27^. The effective strain *u*_*eff*_ in films at a temperature *T* varies with thickness *h* as^26,27^:

1 $${u}_{eff}(T)=u(T)-\frac{u({T}_{G}){a}_{F}(T)}{{a}_{F}({T}_{G})}\beta (1-\frac{{h}_{c}}{h})$$

where *u(T*) and *u(T*_*G*_) is the misfit strain at temperature *T* and at growth temperature *T*_*G*_, *a*_*F*_*(T*) and *a*_*F*_*(T*_*G*_*)* is the film lattice constant at *T* and *T*_*G*_ temperatures, and *h*_*c*_ is the critical film thickness related to the generation of dislocations. We have inserted an empiric correction factor β = 0.5 to take into account that at thicknesses of about 160–175 nm there is still a substantial effective strain, oscillating around 1.5–2% in the film, as previously measured by Geometrical Phase Analysis (GPA) microstrain analysis on HRTEM^16^. In order to obtain the misfit strain at growth temperature (T_G_ = 700 °C), the lattice constants of the substrate (a_S_) and BCTZ film (a_F_) have been calculated by taking into account the room temperature bulk values (a_S_ = 3.905 Å, a_F_ ≈ 4.02 Å) and the linear thermal expansion coefficient α (α_S_ ≈ 10^−5^ K^−1^, α_F_ ≈ 1.2×10^−5^ K^−1^). The critical film thickness above which a greater number of dislocations occurs has been taken about 14 nm^26^. The obtained results for u_eff_ are displayed in the bottom plot in Fig. 6b. It can be observed that the effective strain decreases rapidly with film thickness, but remains above 1% even at 400 nm.

**Figure 7:**
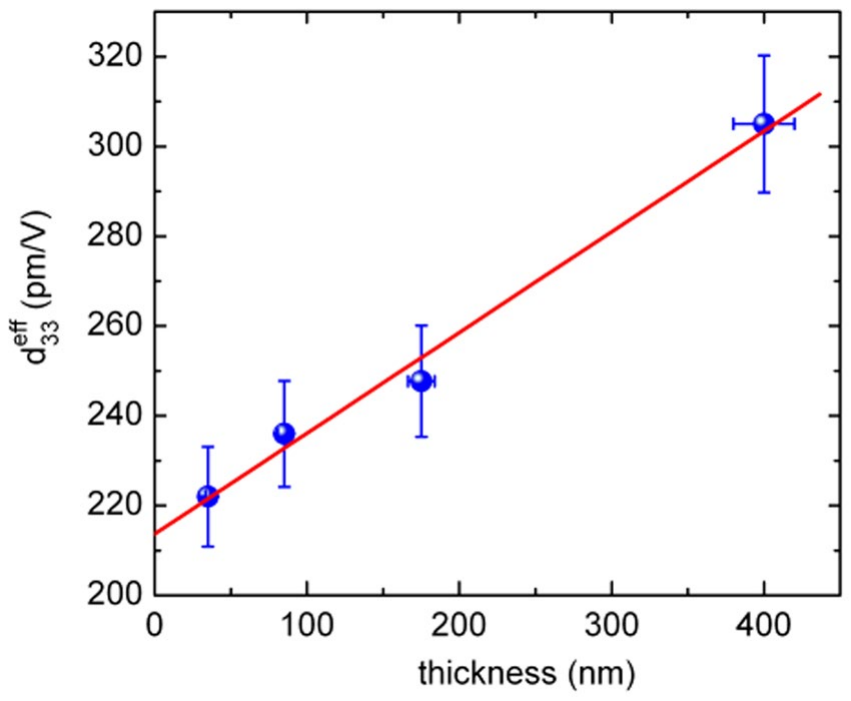
Variation of the effective piezoelectric constant with film thickness.

In contrast with the dielectric behavior evolution with film’s thickness, the local piezoelectric characterization performed with PFM technique shows a more linear trend of the effective piezoelectric coefficient d_33_^eff^ values with films thickness, as can be observed in Fig. 7. For epitaxial thin films it is known that generally piezoelectricity decreases with the decrease of film thickness due to different intrinsic and extrinsic phenomena associated with defects, misfit strain and clamping of the substrate, quality of the electrodes and depolarization fields etc. These effects intervene in a sophisticated manner, through dielectric permittivity, polarization, effective electric field etc, but can be roughly understood in the following simplified approach.

For an epitaxial thin film clamped on the substrate we consider the in-plane strain uniform and equal to the misfit strain. In the absence of extrinsic effects from the electrodes and depolarization fields, the total elastic strain S_3_ in the vertical direction is related to the applied electric field E through the effective piezoelectric coefficient d_33_^eff^ which is given by the following relation^28^:

2 $${d}_{33}^{eff}=\frac{d{S}_{3}}{dE}=2{Q}_{eff}(P+{\varepsilon }_{E}E)({\varepsilon }_{E}+{\varepsilon }_{S})$$

where P is the spontaneous polarization of the ferroelectric, ε_S_ is the spontaneous dielectric permittivity $({\varepsilon }_{S}=\frac{\partial P}{\partial E})$ i.e the dielectric permittivity related to the spontaneous polarization and measured under zero external field, ε_E_ is the field-dependent dielectric permittivity $({\varepsilon }_{E}=\frac{\partial {P}_{E}}{\partial E})$ related to the polarization induced by the applied electric field P_E_ and Q_eff_ is the effective electrostrictive coefficient defined as

3 $${Q}_{eff}={Q}_{11}-\frac{2{s}_{13}{Q}_{12}}{{s}_{11}+{s}_{12}}$$

In the above relationship Q_11_ and Q_12_ are the electrostrictive constants, while s_ij_ are the elastic compliances.

As long as the epitaxial film can be considered rigidly clamped to the substrate, d_33_^eff^ remains much smaller than the bulk values, since the second term in Eq. (3) is negative (s_13_ < 0 and Q_12_ < 0) and decreases Q_eff_. Although the electrostrictive constants in Eq. (3) are not known, we can try an evaluation by using the corresponding values for the basic composition BaTiO_3_: Q_11_ = 0.11 m^4^ C^−2^, Q_12_ = −0.045 m^4^ C^−2 ^^29^. The elastic compliances for the BCTZ 45 composition have been measured in ref.^9^: s_11_^E^ ≈ 13×10^−12^ m^2^/N, s_12_^E^ ≈ s_13_^E^ ≈ −4×10^−12^ m^2^/N. With these values the ratio of the piezoelectric coefficients of a free thin film to a completely clamped film, excluding other contributions, becomes

4 $${d}_{33}/{d}_{33}^{eff}\propto {Q}_{11}/{Q}_{eff}\cong 1.57$$

Thus basically, if we assume that the BCTZ film with h = 35 nm is completely clamped, its piezoelectric constant d_33_^eff^ = 222 pm/V should be related to the free piezoelectric constant value (which we take to be d_33_ ≈ 350 pm/V, corresponding to the bulk BCTZ 45 composition as in ref.^1^) by the Eq. (4). Indeed, their ratio fulfils this relation, showing that although the BCTZ 45 film is very thin, its quality is very high and no extrinsic effects, other than the clamping on the substrate, downsize its piezoelectric properties. Actually in this rough evaluation we have excluded the dependence of spontaneous polarization and permittivity on the internal strain induced by the crystallographic nanodomains, which gives important contributions in the strained thin films. Indeed as it can be observed in Fig. 6b, both the measured permittivity and the tetragonality c/a (which is related to the spontaneous polarization) are higher in the thinnest film, which can eventually compensate for extrinsic effects, if present, and explain the high value of its piezoelectric constant. In the same time, one must take into account the phase structure, which has been shown to strongly affect the electrical properties of lead-free piezoelectric materials^30^. Indeed, the phase coexistence seems to be the key to obtain maximization of functional properties, as evidenced in ref.^30^. The same strategy for construction of multiple phase boundaries (R-O, O-T or R-T) near room temperature is specific also for BCTZ compositions, which for x = 0.5 display a R-T phase boundary with an intermediate O phase, as mentioned in the introduction. However, as pointed out in ref.^30^, there are considerable challenges in attempting to construct phase boundaries in lead-free ferroelectric thin films by using the phase boundary compositions of the corresponding bulk materials. In our case, strain engineering^31^ has been employed to transform the structure of the thin epitaxial BCTZ films from the R to the T symmetry. In this way the electrical properties are increased *via* the polarization enhancement related to the higher tetragonality. Moreover, when the effects of this mechanism decrease due to strain relaxation, another mechanism, associated with the presence of phase boundaries, operates in thicker films. Indeed the structure of the film material free of strain constraining returns R (on average), as in the bulk material. However, at microscopic level the presence of nanodomains with different symmetries was evidenced^16^. It is this nanoscale structure of phase boundaries which could be responsible for the increase of electrical properties in thicker films.

Moreover, it has been pointed out^8,9^ that the maximization of piezoelectric response in BCTZ compositions at MPB (x = 0.5) was achieved at the T-O boundary, due to easier polarization rotation and larger lattice softening (i.e. large compliance constant). The large compliance allows the obtaining of a high response under small force signal. However, one must take into account that BCTZ thin films clamped on the substrate can have a different compliance, and their response can be strongly affected by the conditions of mechanical constraining.

The gradual increase of d_33_^eff^ with film thickness observed in Fig. 7 is nevertheless partially related to the gradual relaxation of the substrate effect which makes piezoelectric constant to approach the free film value. In the same time, one must take into account that these films are still clamped on the substrate, therefore other phenomena could contribute to the increasing of piezoelectric constant as well as to the dielectric permittivity. These include the observed and previously reported^16^ phenomenon of nanoscale phase fluctuations. Briefly it consists in a nanoscale mixing of orthorhombic and tetragonal nanodomains due to the partial relaxation of the tetragonal structure induced by the epitaxial strain from the original rhombohedral structure of the BCTZ 45 composition^16^. We have previously proposed as possible origin of the high dielectric response the nanodomains boundaries contribution. The same phenomenon could also induce a high piezoelectric response to an applied electrical field in thin films, as it was demonstrated for bulk BCTZ by Yang *et al*.^32^. They showed that the physical origin for large piezoresponse occurring in BCTZ compositions is related with the low energy barrier along the minimum energy pathway on the free energy surface for direct domain switching and polarization rotation at the low-symmetry ferroelectric phases boundaries (tetragonal-orthorombic phase boundary). The energy barrier was used to quantitatively measure the degree of polarization anisotropy and piezoelectric properties.

## Conclusions

In summary we have shown that the structural, dielectric and piezoelectric properties of BCTZ 45 epitaxial thin films depend on the thickness in a complex manner. While the bulk material with the same composition has rhombohedral symmetry at room temperature, very thin films show tetragonal symmetry, due to the misfit strain. The effective misfit strain relaxes toward higher thickness, inducing significant changes in tetragonality ratio and dielectric permittivity. However the films remain epitaxial even at the highest thickness of 400 nm. The obtained dielectric permittivity values are increased for the very thin films, due to the increasing of the lattice anisotropy. Very high values are measured also on the thicker films, where the lattice softening allows a higher dielectric response. BCTZ epitaxial thin films show high piezoelectric response for all thicknesses, although there is a decreasing for very thin films due to the clamping on the substrate.

## Methods

Epitaxial thin films have been grown by pulsed laser deposition (PLD) from ceramic targets with composition Ba(Ti_0.8_Zr_0.2_)O_3_-0.45(Ba_0.7_Ca_0.3_)TiO_3_ (BCTZ 45). The ceramic targets have been prepared by conventional solid-state method as previously described^16^. The deposition conditions have been carefully chosen in order to assure epitaxial growth. During the PLD process the pressure of oxygen gas flowing in the deposition chamber was 0.1 mbar. The laser wavelength was 193 nm and the laser fluency was 2.5–2.6 J/cm^2^. The PLD deposition process description and detailed experimental parameters have been previously presented^16^. In this work,the number of laser pulses was varied so that films with various thicknesses in the range 35–400 nm have been obtained. BCTZ 45 thin films have been grown on SrTiO_3_ monocrystalline substrates (001)-cut, heated at 700 °C (for PFM measurements the substrate used was Nb:SrTiO_3_ (STON)). The thickness of the films was measured by spectrometric ellipsometry and confirmed by TEM in cross section.

The structural characterization has been made by conventional and off-axis XRD with a Panalytical X’pert MRD system equipped with Pixcel detector, by using a parallel monochromatic beam (λ Cu Kα1 = 1.540598 Å) provided by a hybrid asymmetric monochromator 2xGe(220).

Cross-section specimens for HRTEM have been obtained by mechanical grinding and ion milling with a Gatan PIPS installation at 4 kV acceleration voltage and 7° beam incidence angle. The samples have been investigated on a JEOL ARM 200 F TEM operated at 200 kV.

**Figure 8:**
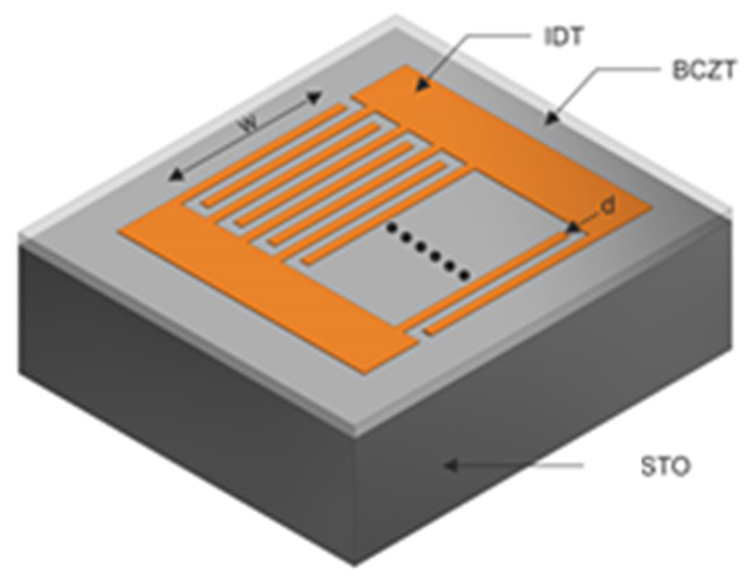
Schematic drawing of the IDTs used for the dielectric characterization.

Interdigital transducers (IDTs) were fabricated on BCTZ film surface in order to perform the dielectric characterization of the deposited layers. The electrodes were made of Au film (100 nm thick) deposited by radio frequency (rf) magnetron sputtering technique, using a commercial MRC 8620 system, from a 99.9% pure Au target. During deposition, the pressure in the chamber was fixed at 3 mTorr with a constant flow rate of 90 sccm of Ar, and with a rf power of 200 W. The obtained deposition rate was 0.087 nm/s and the thickness of the film was estimated measuring the deposition time. The patterns were transferred by a photolithography process, using poly(methyl methacrylate) (PMMA) resist exposed to deep UV radiation, and lift-off procedure. Each IDT consists of 15 couples with a finger width of 7.5 μm (d) and a metallization ratio of 0.5. The finger overlap is of 1.2 mm (w). The schematic drawing of the IDTs used in this work is shown in Fig. 8. At least 10 IDTs have been deposited on the surface of each film, in order to have reliable measurements. After the fabrication, the samples were mounted on a 18 pins microwave package and each IDT was electrically connected by an ultrasonic bonder with Al wires (diameter of 25 μm).

The capacitance and dielectric loss have been measured at different frequencies by using an HP4194A impedance analyser.

The local piezoelectric properties of the BCTZ thin films were investigated by PFM on a commercial AFM (XE-100, Park Systems), by employing a low stiffness (∼1 N/m) titanium-platinum-coated cantilever (NSC36 series, Mikromasch). In order to achieve a good electrical contact, the samples were glued to stainless steel disks with silver paint. The STON substrate acted as bottom electrode while the conductive AFM tip as top electrode. The DC bias was applied to the substrate and the testing AC signal was applied to the tip, both referenced to a common ground. The local out-of-plane mechanical response of the material to the driving signal was recorded during ramping of the DC bias. The amplitude and phase of the piezoelectric out-of-plane response were extracted from the AFM signal by a lock-in amplifier (Stanford Research Systems SR830).

## Electronic supplementary material

Supplementary Information
