# Supplementary material for: Impact of thickness variation on structural, dielectric and piezoelectric properties of (Ba,Ca)(Ti,Zr)O3 epitaxial thin films
Source: Sci Rep. 2018 Feb 1;8:2056. doi: 10.1038/s41598-018-20149-y (PMC5794999; doi:10.1038/s41598-018-20149-y)
Supplement: Supplementary file 1 — Supplementary Information [file 41598_2018_20149_MOESM1_ESM.pdf]

# Supplementary Information

# Impact of thickness variation on structural, dielectric and piezoelectric properties of $(Ba,Ca)(Ti,Zr)O_3$ epitaxial thin films

**Valentin Ion[1], Floriana Craciun[2], Nicu D. Scarisoreanu[1*], Antoniu Moldovan[1], Andreea Andrei[1], Ruxandra Birjega[1], Corneliu Ghica[3], Fabio Di Pietrantonio[4], Domenico Cannatà[4], Massimiliano Benetti[4], Maria Dinescu[1]**

[1] *National Institute for Laser, Plasma and Radiation Physics, 409 Atomistilor, 077125 Magurele, Romania*

[2] *CNR-ISC, Istituto dei Sistemi Complessi, Area della Ricerca di Roma-Tor Vergata, Via del Fosso del Cavaliere 100, I-00133, Rome, Italy*

[3] *National Institute of Materials Physics, 105 bis Atomistilor, 077125 Magurele, Romania*

[4] *CNR-IDASC, Istituto di Acustica e Sensoristica "O. M. Corbino", Via del Fosso del Cavaliere 100, I-00133, Rome, Italy*

E-mail: nicu.scarisoreanu@inflpr.ro

**<u>Content</u>**

Morphologic analysis performed by Atomic Force Microscopy on BCTZ thin films with different thickness.

Structural difference details between BCTZ thin films with different thicknesses.

Compositional homogeneity EDS measurements performed both in TEM and STEM modes

**Morphologic analysis performed by Atomic Force Microscopy on BCTZ thin films with different thickness.**

The AFM analysis has been performed in non-contact mode using an Park System XE-100 with Silicon cantilever. The images of the surface morphologies for films with different thickness are presented in Figure S1 a), b) c) and d).

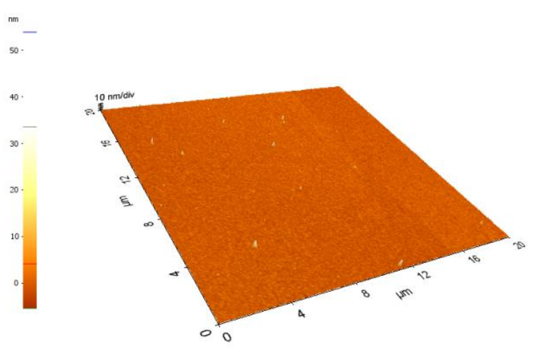


a)

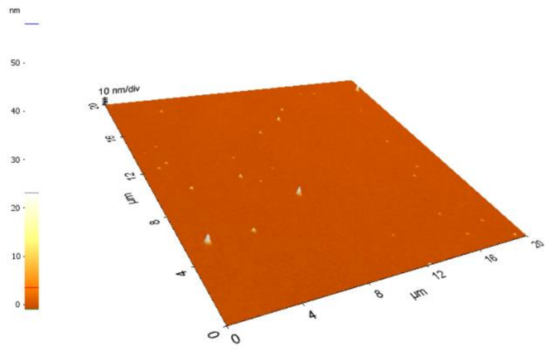


b)

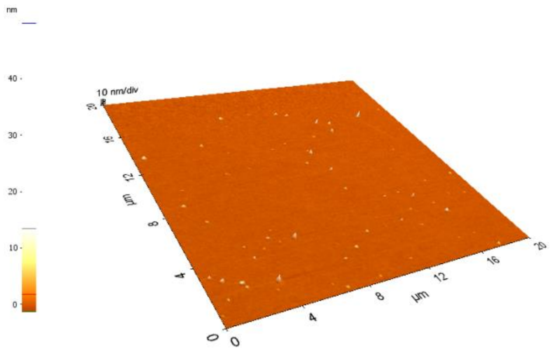


c)

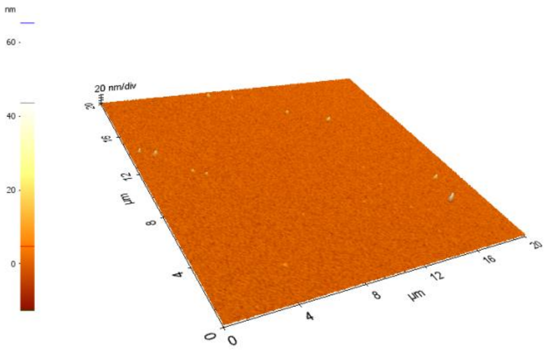


d)

Figure S1. The AFM images of the surface morphologies for BCTZ thin films with different thicknesses: a) 35 nm, RMS-1.7 nm, b) 85 nm, RMS-0.8 nm, c) 175 nm, RMS-0.7 nm, d) 400 nm, RMS-2.2 nm.

**Structural difference details between BCTZ thin films with different thicknesses.**

In Figure S2 a) and b), the enlarged angular regions of the XRD patterns of BCTZ thin films with different thicknesses, around (002) and (004) peaks, respectively are presented.

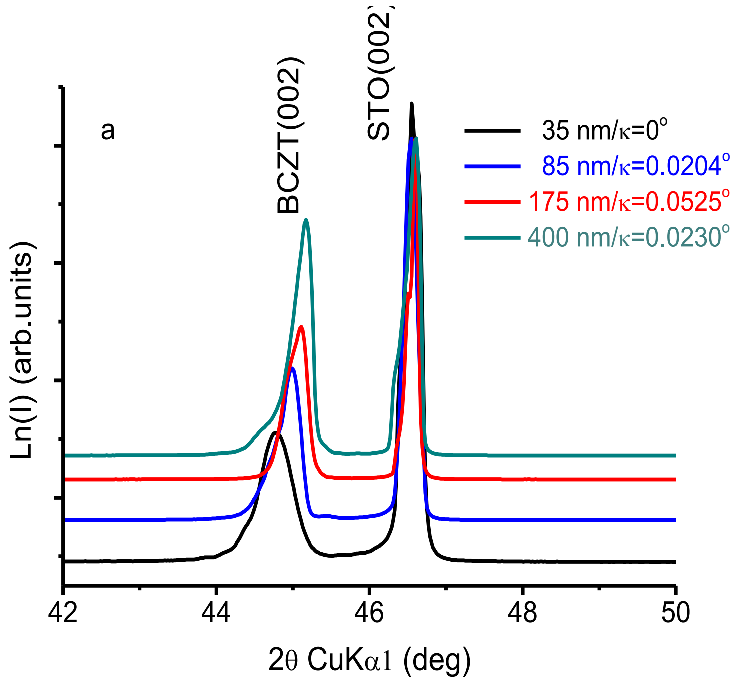


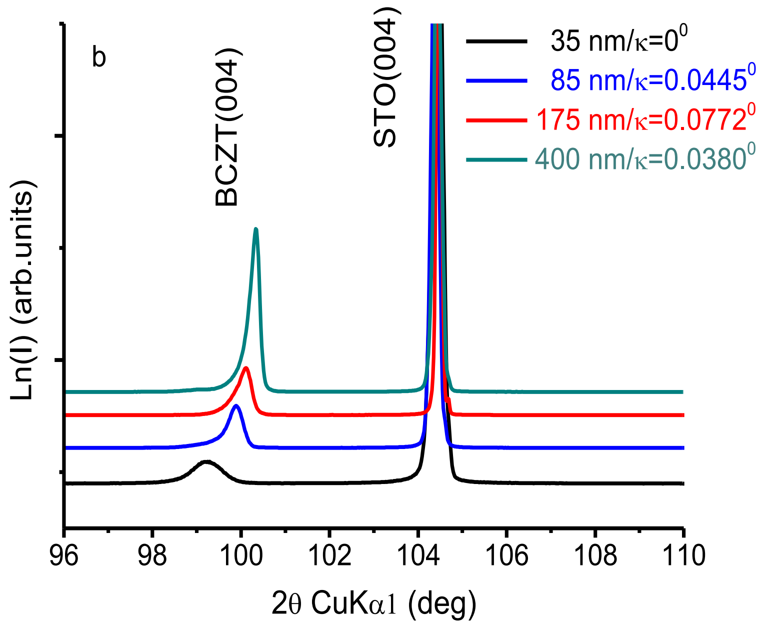


Figure S2. The XRD patterns of the BCZT films in the (002) a) and (004) b) reflections domains. The shift in the asymmetry included in figures was quantified by the parameter κ $=2\theta_{max}-\theta_{mean}$ , were $2\theta_{max}$ and $\theta_{mean}$ are the Bragg-angles of the maximum and the mean of the profile, respectively [1].

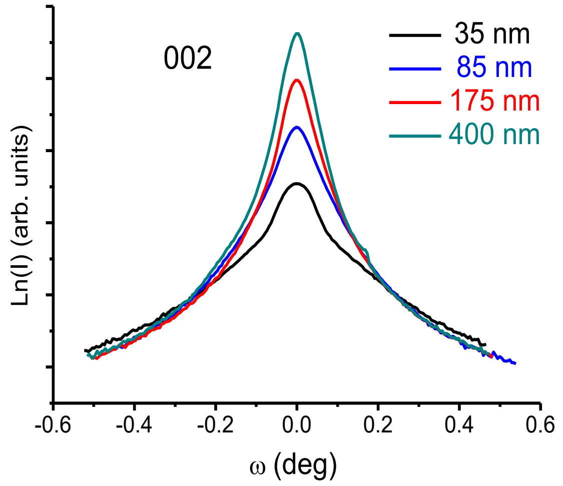


Figure S3. Rocking curves in logarithmic scale of the (002) reflections of the BCZT films.

**Compositional homogeneity EDS measurements performed both in TEM and STEM modes**

The films compositions with respect to the nominal values in the PLD target and the compositional homogeneity of the thin films have been investigated by EDS measurements performed both in TEM and STEM modes. A typical EDS spectrum obtained on a cross-section sample in the TEM mode on the 85 nm thick BCTZ layer using a spot size of the same order of magnitude as the film thickness is presented in figure S5.

.

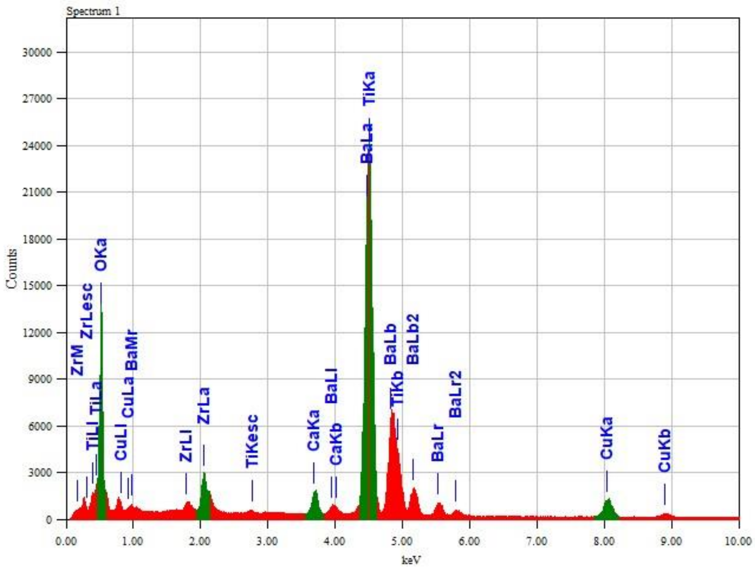


Figure S4. Typical EDS spectrum on the BCTZ layer by cross-section TEM.

The STEM ADF image and elemental line profile across the BCTZ layer and de STEM EDS elemental mapping are presented in figure S5.

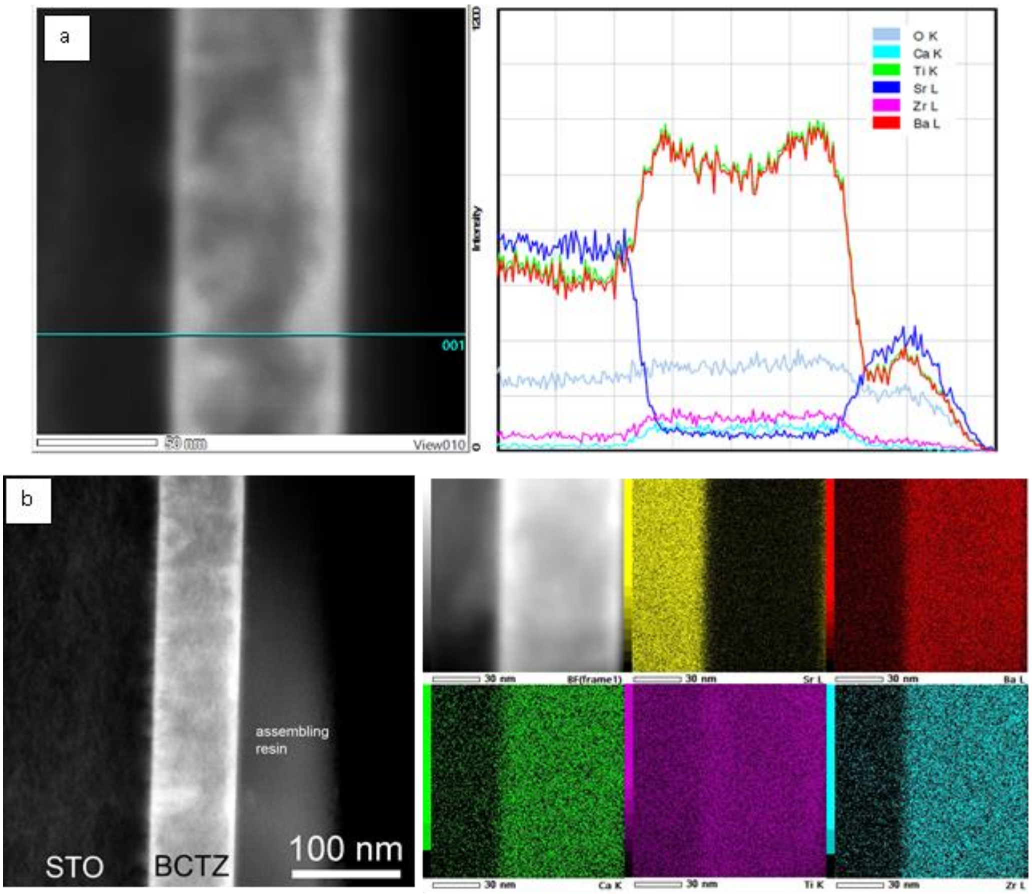


Figure S5. (a) STEM-ADF image and elemental line profile across the BCTZ layer; (b) STEM-ADF image and STEM-EDS elemental mapping of the BCTZ/STO sample.

## *References*

1. Wejdemann, C., Lienert, U., Pantleon,W. Reversal of asymmetry of X-ray peak profiles from individual grains during a strain path change. Scr. Mater. 62, 794-797, (2010)
